# Protein structure prediction by all-atom free-energy refinement

**DOI:** 10.1186/1472-6807-7-12

**Published:** 2007-03-19

**Authors:** Abhinav Verma, Wolfgang Wenzel

**Affiliations:** 1 Institute for Scientific Computing, Forschungszentrum Karlsruhe, Karlsruhe, Germany; 2 Institute for Nanotechnology, Forschungszentrum Karlsruhe, Karlsruhe, Germany

## Abstract

**Background:**

The reliable prediction of protein tertiary structure from the amino acid sequence remains challenging even for small proteins. We have developed an all-atom free-energy protein forcefield (PFF01) that we could use to fold several small proteins from completely extended conformations. Because the computational cost of de-novo folding studies rises steeply with system size, this approach is unsuitable for structure prediction purposes. We therefore investigate here a low-cost free-energy relaxation protocol for protein structure prediction that combines heuristic methods for model generation with all-atom free-energy relaxation in PFF01.

**Results:**

We use PFF01 to rank and cluster the conformations for 32 proteins generated by ROSETTA. For 22/10 high-quality/low quality decoy sets we select near-native conformations with an average C_*α *_root mean square deviation of 3.03 Å/6.04 Å. The protocol incorporates an inherent reliability indicator that succeeds for 78% of the decoy sets. In over 90% of these cases near-native conformations are selected from the decoy set. This success rate is rationalized by the quality of the decoys and the selectivity of the PFF01 forcefield, which ranks near-native conformations an average 3.06 standard deviations below that of the relaxed decoys (Z-score).

**Conclusion:**

All-atom free-energy relaxation with PFF01 emerges as a powerful low-cost approach toward generic de-novo protein structure prediction. The approach can be applied to large all-atom decoy sets of any origin and requires no preexisting structural information to identify the native conformation. The study provides evidence that a large class of proteins may be foldable by PFF01.

## Background

The development of reliable methods for de-novo protein structure prediction remains a challenge [1-4] even for small proteins. Heuristic methods, which dominate protein structure prediction contests [3], can generate accurate models [5], but often lack the ability to reliably identify near-native conformations [6]. Folding simulations using accurate biophysical models demonstrate agreement with experimental investigations [7-11], but remain limited to small proteins by their large associated computational cost. We have developed an all-atom free-energy forcefield [12] to describe the protein folding process. In our folding studies we exploit the thermodynamic hypothesis [13], which stipulates that many proteins in their native configuration are in thermodynamic equilibrium with their environment. Based on this paradigm the native conformation of a protein can be predicted as the global optimum of its free energy surface [14]. Since the free-energy landscape of naturally occurring proteins is thought to have a funnel-like shape [15,16], stochastic search methods are guided by the overall gradient towards the global optimum of this landscape. Using a variety of different stochastic optimization methods we were able to demonstrate the reproducible and predictive folding of several proteins, including the trp-cage protein (1L2Y) [17], the villin headpiece [18], the HIV accessory protein (1F4I) [19], and the bacterial ribosomal protein L20 [20,21] with 20, 36, 40 and 60 acids respectively.

While these studies demonstrate the feasibility of all-atom protein structure prediction from random initial conformations, the numerical effort for a predictive simulation still increases steeply with system size. The numerical effort for a predictive simulation increases from about 20 CPU days (on standard off-the-shelf hardware) for a protein with 20 amino acids to about 8000 CPU days for 60 amino acids [21,22]. In an alternative, widely pursued approach [23-27], protein structures are assembled de-novo according to heuristic principles, such as local sequence homology [28] and then ranked with either knowledge based or forcefield based scoring functions [29-37]. Heuristic decoy generation eliminates the need to the sample the entire conformational space of the protein or to reconstruct the folding pathway. Because large decoys sets of protein-like conformations can be generated much faster than by sampling the free-energy landscape, the decoy selection approach makes it possible to predict the native conformation of proteins that are too large to be folded from completely random initial conformations. Of particular interest in this regard are decoy sets that are generated from a completely orthogonal philosophy from folding, e.g. methods that assemble the protein from fragments obtained from local homology or other sources [28,38,39].

The goals of this investigation are therefore twofold: first test the accuracy of the free-energy forcefield PFF01 for proteins that are too large and too complex to fold from random initial conformations. If we find near-native decoys are lower in free-energy than all other conformations, the forcefield is accurate enough to fold the protein. Since it is impossible to generate completely exhaustive decoy sets we use 32 proteins of the latest ROSETTA all-atom decoy library as a reference [6]. These decoy sets were generated specifically for the purpose of forcefield-assessment and help us to obtain an unbiased assessment of the "universality" of the forcefield.

Secondly we develop and validate a protocol, free-energy relaxation, to select the native protein structure from large libraries of protein conformations generated by a heuristic method. Free-energy relaxation could be used for protein structure prediction either as a stand-alone method, or as a post-scoring approach for existing techniques. Because no fundamentally new conformations are generated in the relaxation protocol, a prerequisite for success is the existence of some near native conformations in the decoy set. This investigation deals only with the validation of a suitable selection protocol, not with the generation of exhaustive decoys sets. Our approach will therefore fail for proteins where the decoy set contains an insufficient number of near-native conformations. An overall assessment of the viability of the free-energy relaxation approach for protein structure prediction would additionally require an independent assessment of the likelihood of the decoy-generator to propose near-native decoys.

## Results

We investigated 32 small proteins (30–85 amino acids) without any stabilizing ligands [6] (see Methods). The proteins have all-alpha (20), alpha-beta (8) or only beta (4) secondary structure and cover many distinct structural families. Previous investigations ranked the decoys and found significant enrichment by several independent descriptors (Lennard-Jones, Coulomb, Hydrogen Bonding, etc) with Z-score ranging from -1 to -2 [6,36]. The Z-scores using the original ROSETTA energies were reported to be poor, indicating that the development of a scoring function to select near-native decoys from this set poses a significant challenge [40-43].

The lowest Z-scores were reported for side-chain Lennard Jones interactions, favoring compact structures and side-chain hydrogen bonding. Neither main-chain hydrogen bonding, nor Coulomb interactions, nor a wide range of implicit solvent models resulted independently in very low Z-scores [6]. The PFF01 forcefield [12] integrates exactly such components (Lennard Jones, electrostatic model, hydrogen bonding, SASA implicit solvent model) but balances them in a fashion that was demonstrated to be highly selective for at least some small proteins. Here we investigate the question whether this unique combination is transferable to a larger protein test set and able to select near-native conformations of these independently generated decoy sets. Use of all applicable decoys of a protein library generated by an alternate approach ensures that the investigation is not biased towards proteins particularly amenable to relaxation with PFF01.

### Decoy ranking

**Figure 1:**
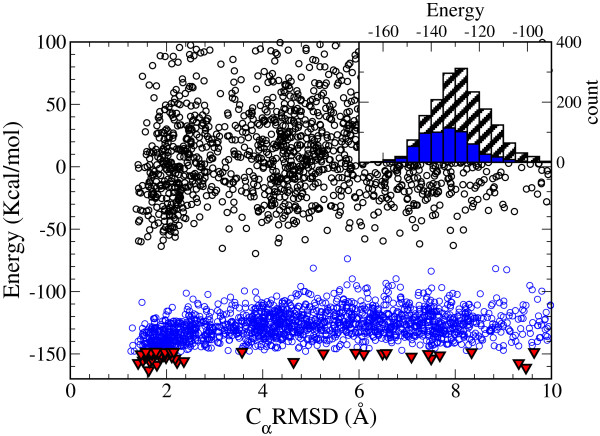
**Energy relaxation**. Each conformation in a decoy set (1r69) is relaxed from its starting conformation (top set) to the final conformation (bottom set), dashed lines correlate the starting and final conformations for a subset of the relaxation runs. The native conformation (red triangle) has the lowest energy and clustering of the fifty lowest energy decoys (blue triangles) predicts the native conformation. Inset: Energy relaxation leads to an enrichment of the near native decoys in the entire set of final conformations(blue set = 50 lowest energy decoys).

Free-energy relaxation scores the decoys in the set according to their energy in the forcefield PFF01 without major structural changes. Since this approach can only succeed for decoy sets containing near-native conformations we have subdivided the protein targets into two families: 22 high-quality decoy sets containing at least 10% near-native conformations and 10 low-quality decoy sets which contain few or no near-native decoys. Throughout this study we define near-native conformations as those with a C_*α *_root mean square deviation (C_*α*_RMSD) of less than 4.5 Å to the native conformation, commensurate with the characteristic resolution of the decoys [6] (less then 1% of the decoys of the low-quality decoy set have a C_*α*_RMSD of less then 4 Å). This measure is commensurate with the quality of the near-native conformations that we find in our folding studies [21,44], which typically converge to about 3–4 Å C_*α*_RMSD, owing to the use of an implicit solvent model. Implicit solvent models, which are required to estimate the solvent contribution to the free energy of a protein conformation, tend to degrade the accuracy of the simulated native ensemble in comparison to explicit water simulations. As a result, we cannot expect the resolution of the forcefield to be better than 3–4 Å C_*α*_RMSD in the present relaxation protocol. Conformations generated from all-atom models are not trivially transferable from one theoretical model to another. In order to obtain a meaningful energy estimate each of the decoys must be relaxed in the new forcefield to a nearby local minimum. We pursued a low-cost approach (see Methods), which places the emphasis on the quality of the initial decoy, rather than on the generation of long trajectories that independently sample the conformational space. In such a rapid energy relaxation decoys will not move far from the starting configuration, but will significantly change their energy (Figure 1). The relaxation process leads to a reordering of the decoys and a substantial enrichment of the low-energy subset with near-native decoys. Due to the stochastic nature of the annealing process the final energy of each of the decoys samples a probabilistic distribution in energy and C_*α*_RMSD, the lowest energy decoy must not be a near-native one (see inset).

For 18 of the 22 high quality decoy sets near-native conformations rank among the 10 best conformations (out of approximately 1900 for each protein); for ten proteins the native conformation is selected solely on the basis of its energy (Table 1). Even for the proteins with non-native lowest energy decoys the low-energy ensemble is significantly enriched with near-native conformations (Table 2). A failure to find a native decoy as the lowest energy conformation can have two sources: either it is a failure of the free-energy model/scoring function to identify the correct structure as the global optimum or the near native ensemble was not properly probed in the decoy generation.

### Forcefield selectivity

**Figure 2:**
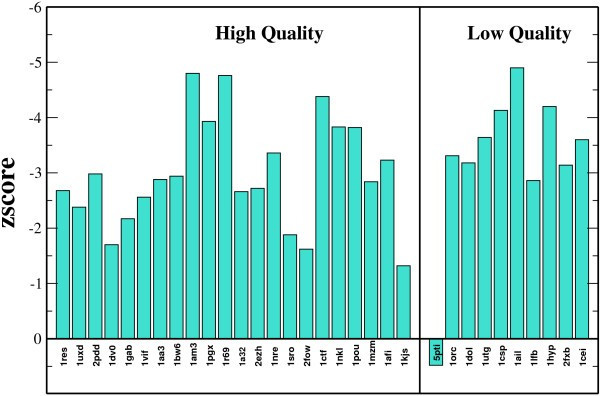
**Z-scores**. Z-scores for independently generated near-native conformations of of the proteins investigated. The Z-score is computed as the ratio of the energy difference between the near-native decoy to the mean of the decoy set, divided by the standard deviation of the letter. The relaxed energies were used for mean and standard deviation.

In order to discriminate between these two possibilities, we independently generated near-native conformations starting from the experimental conformation. Their Z-scores (see Methods) average to -2.98/-3.25 for the high-quality/low quality decoy set (Figure 2).

These values are significantly lower than any reported in previous investigations on the same set of decoys using a variety of different scoring methods [6]. The very good values for the decoys indicate that sampling or ranking problems, rather than forcefield accuracy limit the selectivity of free-energy relaxation. 5pti is the only protein for which a positive Z-score was computed, which is explained by the existence of a large unstructured region in the native conformation that is stabilized by three disulphide bridges. Since disulphide bridges are not accounted for in the present version of the forcefield, it is not surprising that the relaxation protocol generates a large number of decoys with better secondary structure and hydrophobic packing. The Z-score is a function both of the quality of the forcefield and of the decoy set. The Z-scores for low-quality decoy sets are lower than those for high quality decoy sets.

### Decoy clustering

We have used a low-cost computational protocol in order to develop a method that can be applied to very large decoy sets. The best energy found in the short relaxation simulations thus depends stochastically on the moves chosen in the course of the simulation. In order to reduce such fluctuations one may either sample longer or generate several independent trajectories. While both methods may be successful they would significantly increase the overall computational cost to produce a statistically reliable reduction of the energy fluctuations.

Alternatively we can exploit the fact that many decoys are available: we reduce the statistical fluctuations associated with a single short relaxation trajectory by clustering the 50 lowest-energy conformations for each of the decoy sets using a hierarchical algorithm [5,6,45,46]. When a unique cluster emerges during this operation (number of decoys in the largest cluster exceed that of the next-largest cluster by at least 20%), we accept the prediction as "decisive", otherwise we rate the simulation as "indecisive". Just as the available experimental methods routinely fail for many proteins due to lacking crystal or signal quality, computational procedures that can 'solve' only a limited number proteins would be very helpful, provided that they contain an inherent measure of the likelihood of success. Here we use the existence of a 'largest cluster' as a predictor for the decisive simulations.

**Figure 3:**
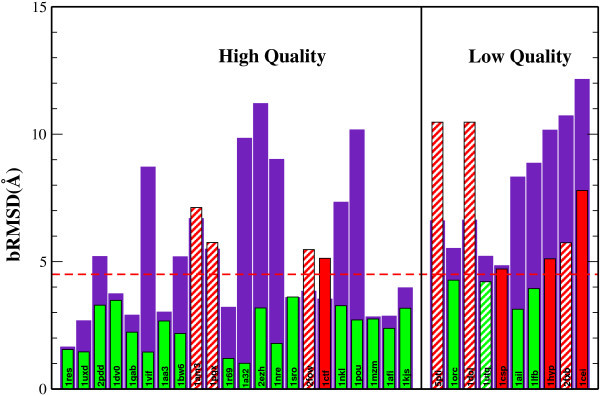
**C_*α*_RMSD of predicted structures**. The C_*α *_RMS deviation between the best cluster and the experimental conformation for the high and low-quality decoy sets (ordered by protein size) shows no decrease of the prediction probability with protein size for the high quality decoy sets. Green bars indicate correct predictions, red bars prediction failures. Shaded bars indicate indecisive simulations (see text), where there was no 'largest cluster' in the top 50 decoys. We note that there is only one genuine prediction failure for the high-quality decoy sets. The horizontal dashed line indicates the acceptance threshold for correct predictions. The purple bars designate the deviations of the best decoys when the same clustering technique is applied with the original ROSETTA scoring function.

**Figure 4:**
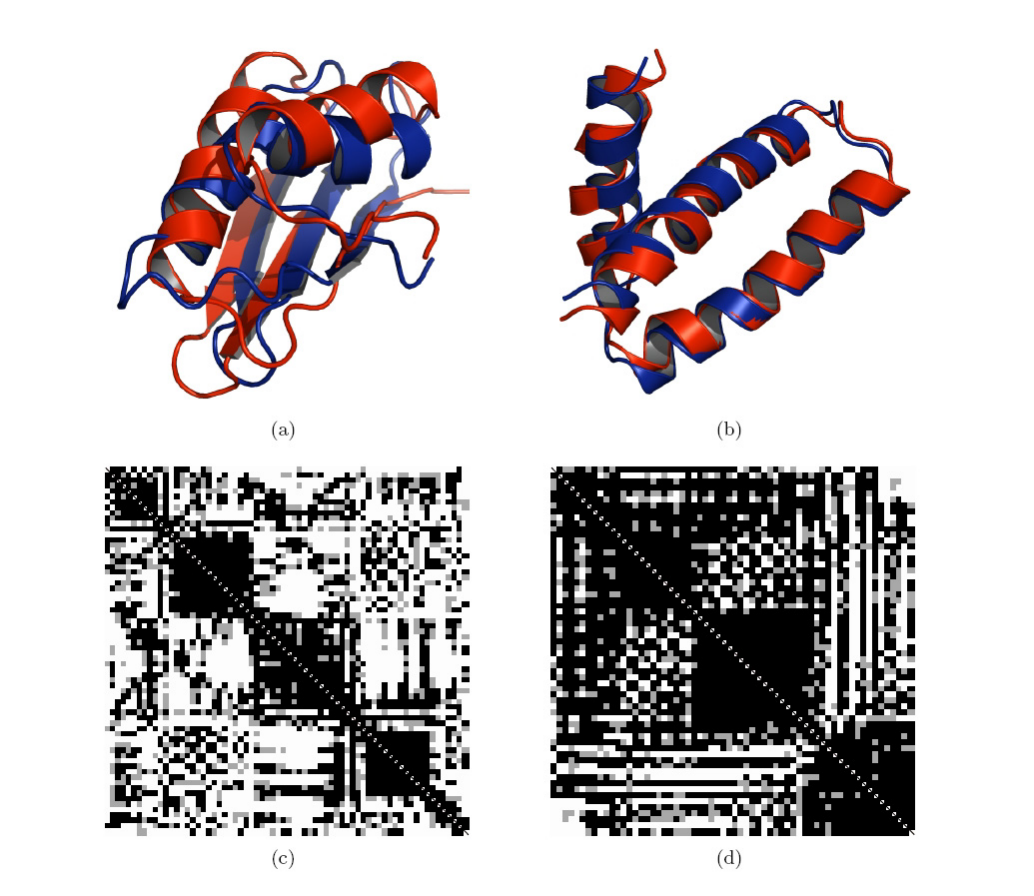
**Top Row: Overlay of some predicted structures. **The overlay of the predicted (red) and experimental conformation (green) documents the close agreement of conformations for 1orc[66] and 1ail[67]; only the backbone of the proteins is shown for clarity (generated with PYMOL [62]). Bottom row:  C_β_-C_β_ distance difference matrices: A pixel in row i and column j of the color coded distance map indicates the difference in the C_β_-C_β_ distances of the native and the folded structure. Black (gray) squares indicate that the C_β_-C_β_ distances of the native and the other structure differ by less than 1.5 (2.25)Å respectively. White squares indicate larger deviations. The data indicates clearly the presence of all short-range and long-range native contacts for the high-quality decoys and good agreement even for the predictions from low-quality decoy sets.

Applying this criterion to the high-quality decoy sets find decisive predictions for 19 out of 22 proteins. For the decisive simulations we predict near-native conformations (left panel of Figure 3) for all but one protein. The single prediction failure (1ctf, C_*α*_RMSD: 5.2 Å) occurred for a dimeric protein. On average the C_*α*_RMSD differs by just 2.4 Å from the experimental conformation. In some cases we approach experimental resolution. Three of the four prediction failures are marked as correctly indecisive, indicating that the prediction protocol is able to differentiate between prediction success and failure based on an inherent criterion. Following this protocol correct predictions are achieved in 90% of the decisive simulations (78% of the proteins). Figure 4 (a)-(c) demonstrates the impressive agreement between the predicted and the experimental conformation for three nontrivial proteins, the presence of the correct secondary structure, stabilizing tertiary contacts and hydrophobic cores.

**Figure 5:**
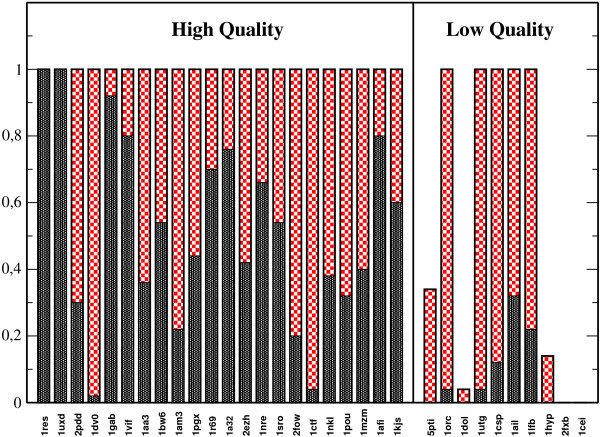
**Enrichment**. Enrichment of native conformations by PFF01: Fraction of near-native conformations in the top 50 decoys by energy (black) and by C_α_RMSD (red). The latter bar indicates the enrichment attainable by an 'ideal' scoring function.

We have applied the same computational procedure to the 10 low-quality decoy sets, which contain few or no near-native conformations (Table 2). Not surprisingly only non-native conformations have the lowest energy for all of these decoy sets, but near-native decoys still rank high in the decoy set. Applying the same clustering technique as above, we obtain correct predictions in three of the six decisive cases. The prediction failure for 1hyp is explained by the presence of four disulphide bridges not accounted for in the model and 1csp is an all-beta protein that is problematic to treat with PFF01. In addition we find one accurate prediction (1utg) that is labeled as indecisive. The quality of the models for representative difficult cases is illustrated in Figure 4 (d)-(f), which demonstrates a still significant similarity of the tertiary structure of the models and the experimental conformations. For most of the low-quality decoy sets differences between the model and the biologically active unit are responsible for the prediction failure. The existence of the many of the correct long range native contacts in the predicted structures is demonstrated in the C_*β*_-C_*β *_distance difference matrices shown in Figure 5. Tertiary contacts are characterized by comparing the difference in distance between pairs of amino acids of two conformations, which correspond to the NOE signals in NMR experiments. In the figure we show the C_*β*_-C_*β *_distance comparison between the model and the experimental conformation for the proteins shown in Figure 4(same order).

## Discussion

**Figure 6:**
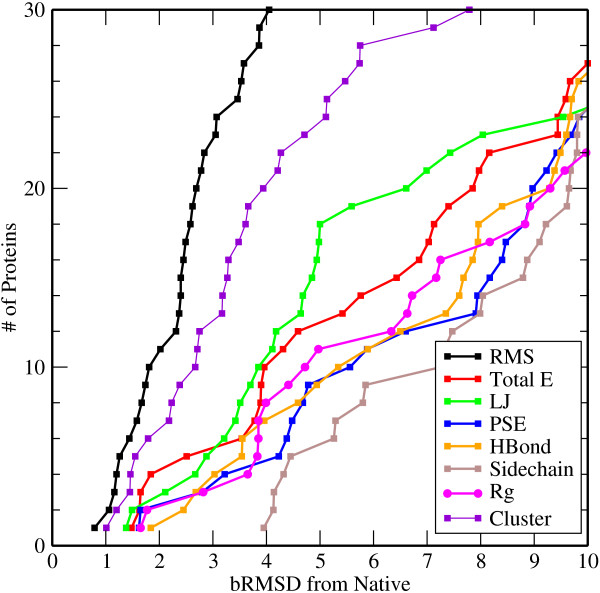
**Enrichment. **Enrichment of the best decoys by C_α_RMSD, total energy in PFF01, and its components (Lennard-Jones, Solvation Energy, Hydrogen Bonding, Sidechain Electrostatics), Radius of Gyration and the clustering technique described in the text. The vertical axis counts the number of proteins in the database that yield a best decoy according to chosen criterion.

In order to put these results into perspective we have investigated the enrichment of near native decoys in each decoy set. We computed the fraction of near native (as defined above) conformations in the top 50 decoys that were used for clustering using the free-energy criterion and the C_*α*_RMSD to the native conformation as ordering criteria (Figure 6). The latter fraction is a measure of the quality of the decoy set: it approaches one when a sufficient number of near native decoys is present. The first fraction is a measure of the selectivity of the free-energy relaxation protocol. Correct predictions are obviously rendered in those cases where all lowest energy decoys are near-native (1res for example) and in those cases, where near-native decoys dominate the top 50 configurations (for example: 1afi, 1gab). The clustering scheme gives acceptable predictions even when only 30% of the low-energy decoys are near-native (for example: 2pdd), but routinely fails when the selectivity of the forcefield is insufficient (1am3). There is clearly room for improvement in the clustering protocol, because some decoys with a relatively large number of top-scoring near-natives nevertheless fail to generate near-native predictions (for example: 1pgx). For the low-quality decoy sets there is a strong correlation between the fraction of good decoys and the success of the approach. Even when the fraction of near-native decoys in the decoy-set drops below 10%, a relatively small number of selected near-natives is sufficient to obtain a near-native prediction. This observation indicates that a search for improved relaxation protocols may help to reduce the required fraction of near-native decoys for a successful prediction below 10% of the overall database.

**Figure 7:**
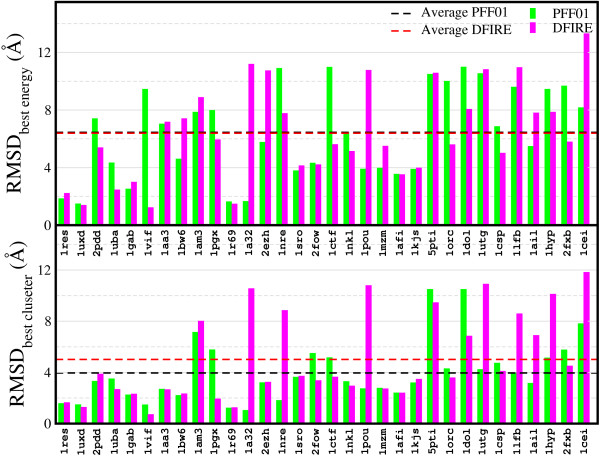
Comparison with DFIRE: RMSD of best energy (top row) and best cluster (bottom row) for the decoy sets by PFF01 (green bar) with DFIRE (red bar). The dashed lines indicate the averages over all decoy sets.

This observation is also supported when we analyze the top decoy of each decoy set by a variety of measures. Following the analysis of Tsai et.al. [6], we show the number of proteins for which the top conformation is selected by various scoring methods (Figure 7). Ranked by C_*α*_RMSD we find that about half of the decoy sets contain at least one decoy with a C_*α*_RMSD of less than 2.5 Å. None of the scoring functions is capable to find this single 'needle in the haystack'. If we look at the error range of 3–4 Å C_*α*_RMSD, which is commensurate with our folding simulations [12,18,19], the relaxation/clustering technique is far superior to any of the other indicators investigated here. The next best scoring function is the Lennard-Jones energy as was reported previously [6]. In comparison, many other indicators that are believed to correlate highly with 'nativeness', such as the existence of secondary structure (as measured by hydrogen bonding energy), solvation terms or sidechain electrostatics, are much less selective. This selectivity of the Lennard-Jones interaction is presently not understood, because the Lennard-Jones energy gives only a small contribution to the overall 'folding' energy in our folding simulations. We hypothesized that Lennard Jones interactions simply measure 'compactness' and ranked the decoys by their radius of gyration as a similar measure. However, the radius of gyration emerged as a much less sensitive measure. The Lennard-Jones energy also does not remove many clashing conformations, because the decoy sets are of very high quality with regard to steric hindrance. The data compilation with the ROSETTA scoring function leads to a highly preselected set of conformations, which might bias the results. Our observation might be explained by the fact that many decoys are already near-optimal with respect to the other energy terms, so that the Lennard-Jones term emerges with higher selectivity than with a set of random conformations. We have repeated the analysis also by analyzing the best C_*α*_RMSD of the top five decoys in each category to reduce possible scatter and find the qualitatively the same results (data not shown). The fact that total energy and clustering technique are by far the most selective indicates that it is the combination of terms in the forcefield which results in the high overall selectivity of the method. This is also confirmed by comparing our results with those of ROSETTA. We have scored all the proteins with the original ROSETTA [28,47-49] scoring function and applied the same clustering techniques as described above (purple bars in Figure 3). We find that the present method leads to a significant improvement of the C_*α*_RMSD for all but one decoy set with decisive predictions. It is therefore the combination of a powerful method for decoy generation in combination with the additional selectivity provided by the all-atom forcefield that generates the high-selectivity of the relaxation approach.

We have also computed the logPB1 and logPB10 values that were used to characterize the selectivity of the density scoring/self-RAPDF function in a recent investigation using the same decoy set [36] (Table 3). We find average values of-0.48 for logPB1 and -1.43 for logPB10 respectively, which compares with -0.92/-1.46 for the density scoring function and -1.0/-1.6 for self-RAPDF for the same subset of decoys. The ranking of native conformations per se is not important for structure prediction since it may not be an indicator of how well a function can select near-native decoys. In other words, it is relatively easy to design functions that discriminate the native conformation from a set of decoys, but hard to design functions that can discriminate near-native decoys from other decoys. This scenario applies exactly here: as Table 3 demonstrates, self-RAPDF works very well to select the best decoys from decoy sets that contain a significant fraction (> 30%) of near-native decoys by our definition, while the present protocol may rank the energetically best decoy comparatively badly in a set of only good decoys. Due to the inherent limitation of its resolution, PFF01 is not a good forcefield for the second purpose, we obtain therefore comparatively bad logPB1/10 values for very good decoy sets. If instead we focus on the selection of near-native decoys from decoys sets with large non-native fractions, the present protocol outperforms self-RAPDF. In Table 3 we have marked the decoy sets where a given method select at least one near native decoy in the top ten. Using this selection criterion, we find that PFF01 succeeds in 78% of the cases, in comparison to 50% for self-RAPDF. The approach pursued here is useful to select low-resolution decoys from complex decoy sets, which contain many non-native competitors, with high probability [50].

## Conclusion

We have investigated a straightforward all-atom energy-relaxation protocol for protein structure prediction. We scored all conformations in a given decoy set using our all-atom forcefield PPF01 after a rapid relaxation procedure, followed by a hierarchical clustering of the 50 top-scoring decoys. We label a relaxation as "decisive" if the lowest energy cluster is at least 20% larger than any other.

With this approach we have succeeded to assign all-atom tertiary structure to 78% of the proteins (marked as decisive) investigated in this study with an average C_*α*_RMSD of 3.12 Å. Exploiting the inherent success criterion of our approach a near-native conformation was predicted in 90% of the decisive relaxation simulations. This high degree of success is rationalized by the high selectivity of the forcefield. We find an average Z-score of-3.03 for independently generated near-native conformations with respect to the decoy sets. PFF01 stabilizes the native conformation of all but one protein against the decoys in the data set. The protocol investigated here has a success threshold of about 10% of native decoys, but appears to succeed at least occasionally for lower native content of the decoy set. Further improvements in the forcefield and the relaxation protocol may be able to push the required fraction of native conformations to even lower values.

The accuracy of the predicted structures appears to be limited 3–4 Å by the resolution of the present free-energy forcefield. This resolution is comparable to that of our folding investigations and commensurate with other folding studies using implicit solvent forcefields [51]. In order to improve the accuracy further, one can either design all-atom explicit water protocols that start from the predicted structures or rank families of near-native conformations by knowledge based scoring functions such as self-RAPDF [36] that are more selective in the near-native conformational space.

Energy relaxation emerges as a powerful low-cost approach (20–50 CPU days in parallel per decoy set) toward generic de-novo protein structure prediction. It can be applied to large all-atom decoy sets of any origin and requires no preexisting structural information to identify the native conformation. We have confined the present investigation to the ROSETTA decoy sets, because the computation of selectivitiy indicators (such as the Z-Score) or the success rate for prediction obviously depend on the methods by which the decoy sets were generated. The ROSETTA decoy set was explicitely generated for forcefield validation with one coherent protocol and thus gives comparable results for a wide range of structurally distinct small proteins. In addition, the protocol investigated here is based on a generic, publically available technique and can thus be used as the basis of a protocol for protein structure prediction in the CASP competition. Other decoy sets (such as decoys-are-US), which contain also larger proteins, will be investigated in future studies. We stress that only one of two important ingredients to protein structure prediction, the ability of the relaxation protocol to select near-native conformations for diverse decoy sets, was investigated here.

## Methods

### Forcefield

The all-atom (with the exception of apolar CH_*n *_groups) free-energy forcefield PFF01 [12] parametrizes the internal free-energy of a protein macro state in a minimal thermodynamic approach [12,19,52]. The forcefield parametrizes the internal free energy of the protein (excluding backbone entropy) and contains the following non-bonded interactions:

V({r→i})=∑ijVij[(Rijrij)12−2(Rijrij)6]+∑ijqiqjεg(i)g(j)rij+∑iσiAi+∑hbondsVhb.     (1)
 MathType@MTEF@5@5@+=feaafiart1ev1aaatCvAUfKttLearuWrP9MDH5MBPbIqV92AaeXatLxBI9gBaebbnrfifHhDYfgasaacH8akY=wiFfYdH8Gipec8Eeeu0xXdbba9frFj0=OqFfea0dXdd9vqai=hGuQ8kuc9pgc9s8qqaq=dirpe0xb9q8qiLsFr0=vr0=vr0dc8meaabaqaciaacaGaaeqabaqabeGadaaakeaafaqaaeGadaaabaGaemOvayLaeiikaGIaei4EaSNafmOCaiNbaSaadaWgaaWcbaGaemyAaKgabeaakiabc2ha9jabcMcaPaqaaiabg2da9aqaamaaqafabaGaemOvay1aaSbaaSqaaiabdMgaPjabdQgaQbqabaaabaGaemyAaKMaemOAaOgabeqdcqGHris5aOWaamWaaeaadaqadaqaamaalaaabaGaemOuai1aaSbaaSqaaiabdMgaPjabdQgaQbqabaaakeaacqWGYbGCdaWgaaWcbaGaemyAaKMaemOAaOgabeaaaaaakiaawIcacaGLPaaadaahaaWcbeqaaiabigdaXiabikdaYaaakiabgkHiTiabikdaYmaabmaabaWaaSaaaeaacqWGsbGudaWgaaWcbaGaemyAaKMaemOAaOgabeaaaOqaaiabdkhaYnaaBaaaleaacqWGPbqAcqWGQbGAaeqaaaaaaOGaayjkaiaawMcaamaaCaaaleqabaGaeGOnaydaaaGccaGLBbGaayzxaaaabaaabaaabaGaey4kaSYaaabuaeaadaWcaaqaaiabdghaXnaaBaaaleaacqWGPbqAaeqaaOGaemyCae3aaSbaaSqaaiabdQgaQbqabaaakeaaiiGacqWF1oqzdaWgaaWcbaGaem4zaCMaeiikaGIaemyAaKMaeiykaKIaem4zaCMaeiikaGIaemOAaOMaeiykaKcabeaakiabdkhaYnaaBaaaleaacqWGPbqAcqWGQbGAaeqaaaaakiabgUcaRmaaqafabaGae83Wdm3aaSbaaSqaaiabdMgaPbqabaGccqWGbbqqdaWgaaWcbaGaemyAaKgabeaaaeaacqWGPbqAaeqaniabggHiLdGccqGHRaWkdaaeqbqaaiabdAfawnaaBaaaleaacqWGObaAcqWGIbGyaeqaaaqaaGqaaiab+HgaOjab+jgaIjab+9gaVjab+5gaUjab+rgaKjab+nhaZbqab0GaeyyeIuoakiabc6caUaWcbaGaemyAaKMaemOAaOgabeqdcqGHris5aaaakiaaxMaacaWLjaWaaeWaaeaacqaIXaqmaiaawIcacaGLPaaaaaa@93CA@

Here *r*_*ij *_denotes the distance between atoms *i *and *j *and *g*(*i*) the type of the amino acid i. The Lennard Jones parameters (*V*_*ij*_, *R*_*ij *_for potential depths and equilibrium distance) depend on the type of the atom pair and were adjusted to satisfy constraints derived from a set of 138 proteins of the PDB database [52-54]. The non-trivial electrostatic interactions in proteins are represented via group- and position dependent dielectric constants (*ε*_*g*(*i*)*g*(*j*) _depending on the amino-acids to which the atoms *i *and *j *belong). The partial charges *q*_*i *_and the dielectric constants were derived in a potential-of-mean-force approach [55] [see Additional file 1]. Interactions with the solvent were first fit in a minimal solvent accessible surface model [56] parameterized by free energies per unit area *σ*_*i *_to reproduce the enthalpies of solvation of the Gly-X-Gly family of peptides [57]. *A*_*i *_corresponds to the area of atom *i *that is in contact with a fictitious solvent. Hydrogen bonds are described via dipole-dipole interactions included in the electrostatic terms and an additional short range term for backbone-backbone hydrogen bonding (CO to NH) which depends on the OH distance, the angle between N,H and O along the bond and the angle between the CO and NH axis [12,58].

In the folding process under physiological conditions the degrees of freedom of a peptide are confined to rotations about single bonds. In our simulation we therefore consider only moves around the sidechain and backbone dihedral angles, which are attempted with thirty and seventy percent probability respectively. The moves for the sidechain angles are drawn from an equidistributed interval with a maximal change of 5 degrees. Half of the backbone moves are generated in the same fashion, the remainder is generated from a move library that was designed to reflect the natural amino-acid dependent bias towards the formation of *α*-helices or *β*-sheets. The probability distribution of the move library was fitted to experimental probabilities observed in the PDB database [59]. While driving the simulation towards the formation of secondary structure, the move library introduces no bias towards helical or sheet structures beyond that encountered in nature.

### Decoy sets and relaxation

The decoy sets were provided electronically by J. Tsai [6], we have excluded decoy sets that contained only fragments of the experimental structure (2ptl,1tuc,1vcc), that contain iron clusters, stabilizing ions or heavy metals not parametrized in our forcefield (1bq9, 1cc5, 1ptq, 1tif, 5icb). 1msi is an antifreeze protein [60], coordinating a shell of crystal water that cannot be described with an implicit solvent model. Each decoy was relaxed in a single simulated annealing run (50,000 steps, T_*start *_= 200 K, T_*final *_= 3 K). The decoys were clustered in a hierarchical algorithm [45]. Near-native conformations were generated in 50 independent basin hopping simulations [61] starting from the native conformation, each comprising 50 simulated annealing cycles with the same protocol as above using a threshold acceptance criterion of 1 kcal/mol.

## Competing interests

The author(s) declare that they have no competing interests.

## Authors' contributions

Simulations and preparation of the manuscript was jointly performed AV and WW. Both authors read and approved the final manuscript.

## Supplementary Material

Additional file 1 PFF01 Electrostratics. Full description of the electrostatic model used in PFF01. Click here for file

## Figures and Tables

**Table 1:** Results for high quality data sets

pdb	nres	<3	3–4	Δ_1best_	Δ_1min_	R	Δ_1cl_	N	Z
1res	35	0.97	0.02	1.84	1.84	1	1.55	21	-2.68
1uxd	43	0.62	0.12	1.48	1.48	1	1.46	31	-2.38
2pdd	43	0.09	0.22	7.40	3.76	10	3.29	43	-2.98
1dv0	45	0.00	0.10	4.33	2.93	7	3.48	31	-1.70
1gab	47	0.37	0.23	2.51	2.51	1	2.23	46	-2.17
1vif	48	0.28	0.02	9.44	2.33	2	1.45	42	-2.56
1aa3	56	0.01	0.17	7.03	3.57	9	2.67	27	-2.88
1bw6	56	0.14	0.21	4.59	3.65	3	2.18	25	-2.94
1am3	57	0.30	0.09	7.85	3.32	14	7.12	18	-4.80
1pgx	57	0.12	0.18	7.97	2.92	7	5.75	28	-3.93
1r69	61	0.22	0.07	1.63	1.63	1	1.20	37	-4.76
1a32	65	0.23	0.08	1.65	1.65	1	1.01	38	-2.66
2ezh	65	0.01	0.17	5.76	3.17	4	3.18	28	-2.72
1nre	66	0.08	0.14	10.89	2.38	2	1.79	38	-3.36
1sro	66	0.00	0.14	3.78	3.78	1	3.61	36	-1.88
2fow	66	0.00	0.12	4.31	3.67	31	5.47	26	-1.62
1ctf	67	0.00	0.19	10.97	3.99	76	5.13	46	-4.38
1nkl	70	0.00	0.14	6.43	3.55	4	3.27	28	-3.83
1pou	70	0.01	0.10	3.90	3.90	1	2.71	30	-3.82
1mzm	71	0.00	0.16	3.96	3.96	1	2.75	39	-2.84
1afi	72	0.02	0.24	3.54	3.54	1	2.38	49	-3.23
1kjs	74	0.00	0.17	3.89	3.89	1	3.17	48	-1.32

Name, number of amino acids, fraction of decoys with less than 3 Å and between 3–4 Å C_*α*_RMSD respectively, D deviation of the energetically lowest/lowest near-native decoy (Δ_1best_/Δ_1min_) and the rank of the latter (A rank of one indicates that the lowest energy decoy is near-native), Δ_1cl _is the C_*α*_RMSD of the largest cluster of the fifty best decoys, followed by the size of this cluster(N) and Z-score of the native decoys against the decoy set.

**Table 2:** Results for low quality data sets

pdb	nres	<3	3–4	Δ_1best_	Δ_1min_	R	Δ_1cl_	N	Z
5pti	55	0.00	0.00	10.48	3.98	718	10.47	16	0.48
1orc	56	0.00	0.09	10.00	3.99	42	4.27	27	-3.31
1dol	62	0.00	0.00	10.98	3.87	0	10.47	22	-3.18
1utg	62	0.00	0.01	10.53	3.96	213	4.21	29	-3.64
1csp	64	0.00	0.02	6.85	3.97	20	4.71	44	-4.13
1lfb	69	0.00	0.04	9.59	2.45	7	3.94	31	-2.86
1ail	67	0.00	0.02	5.42	3.00	6	3.66	22	-4.90
1hyp	75	0.00	0.00	9.44	0.00	0	5.11	25	-4.20
2fxb	81	0.00	0.00	9.67	0.00	0	5.74	29	-3.14
1cei	85	0.00	0.00	8.16	0.00	0	7.79	37	-3.60

Data as in Table 1.

**Table 3:** Ranking and Selectivity

				LogPB1			LogPB10			Selectivity	
pdb	nres	<3	3–4	PFF01	D-Score	Self RAPDF	PFF01	D-Score	Self RAPDF	PFF01	Self RAPDF

1res	35	0.97	0.02	-0.51	-0.56	-0.41	-0.86	-0.57	-0.57	Yes	Yes
1uxd	43	0.62	0.12	-0.70	-1.02	-1.20	-2.43	-1.21	-2.43	Yes	Yes
2pdd	43	0.09	0.22	-0.10	-0.27	-1.01	-0.59	-1.09	-1.56	Yes	Yes
1dv0	45	0.00	0.10	-0.06	-0.74	-0.63	-1.57	-1.22	-2.05	Yes	Yes
1gab	47	0.37	0.23	-0.72	-0.74	-0.64	-1.32	-0.74	-0.70	Yes	No
1vif	48	0.28	0.02	-0.10	-0.91	-0.97	-0.87	-1.03	-0.97	Yes	No
1aa3	56	0.01	0.17	-0.32	-0.68	-0.52	-2.96	-0.77	-0.70	Yes	No
1bw6	56	0.14	0.21	-0.37	-2.22	-1.04	-2.68	-2.50	-2.22	Yes	Yes
1am3	57	0.30	0.09	-0.14	-0.89	-2.38	-0.25	-1.92	-2.38	No	Yes
1pgx	57	0.12	0.18	-0.08	-0.60	-0.37	-1.04	-0.60	-0.47	Yes	No
1r69	61	0.22	0.07	-1.60	-0.90	-1.82	-2.46	-1.03	-1.82	Yes	Yes
1a32	65	0.23	0.08	-1.12	-0.66	-0.97	-1.91	-1.02	-1.41	Yes	Yes
2ezh	65	0.01	0.17	-0.41	-0.57	-0.86	-1.75	-2.24	-1.06	Yes	Yes
1nre	66	0.08	0.14	-0.09	-0.80	-0.94	-1.62	-1.50	-2.68	Yes	Yes
1sro	66	0.00	0.14	-1.26	-0.61	-0.66	-1.26	-0.83	-0.84	Yes	No
2fow	66	0.00	0.12	-0.63	-1.49	-1.25	-0.63	-2.22	-1.39	Yes	No
1ctf	67	0.00	0.19	-0.03	-0.80	-0.27	-0.81	-2.44	-1.64	Yes	No
1nkl	70	0.00	0.14	-0.27	-0.96	-0.96	-1.27	-1.41	-1.03	Yes	Yes
1pou	70	0.01	0.10	-0.88	-0.77	-2.13	-1.03	-2.13	-2.80	Yes	Yes
1mzm	71	0.00	0.16	-0.82	-0.64	-0.32	-1.61	-0.66	-0.85	Yes	No
1afi	72	0.02	0.24	-0.93	-1.85	-2.18	-1.56	-2.22	-2.78	Yes	Yes
1kjs	74	0.00	0.17	-0.88	-1.39	-1.39	-2.43	-1.68	-2.16	Yes	Yes
5pti	55	0.00	0.00	-0.13	-0.50	-0.50	-0.69	-1.28	-1.28	No	No
1orc	56	0.00	0.09	-0.01	-0.74	-0.74	-0.74	-1.09	-1.03	Yes	No
1dol	62	0.00	0.00	-0.12	-1.27	-0.69	-0.57	-1.27	-1.58	No	No
1utg	62	0.00	0.01	-0.11	-0.55	-0.73	-1.38	-0.73	-0.80	Yes	No
1csp	64	0.00	0.02	-0.51	-0.70	-0.58	-0.62	-1.03	-1.47	No	No
1ail	67	0.00	0.02	-0.70	-1.28	-1.77	-2.78	-1.77	-3.26	Yes	Yes
1lfb	69	0.00	0.04	-0.25	-0.98	-1.86	-3.28	-1.65	-2.02	Yes	Yes
1hyp	75	0.00	0.00	-0.36	-0.75	-0.89	-0.53	-1.62	-1.09	No	No
2fxb	81	0.00	0.00	-0.41	-1.49	-0.87	-1.51	-2.30	-2.65	No	No
1cei	85	0.00	0.00	-0.55	-1.01	-1.18	-0.59	-2.80	-2.38	No	No

Comparison of ranking and selectivity of different scoring functions: PDB id, number of amino acids, fraction of decoys with less than 3 Å and between 3–4 Å C_*α*_RMSD respectively. The next columns show the logPB1/logPB10 values for different scoring functions, the data for density score(D-score) and self-RAPDF was taken from [36]. The last two columns indicate whether PFF01 or self-RAPDF were able to select one near-native decoy in the 10 energetically best decoys.
