# Supplementary material for: Protein structure prediction by all-atom free-energy refinement
Source: BMC Struct Biol. 2007 Mar 19;7:12. doi: 10.1186/1472-6807-7-12 (PMC1832197; doi:10.1186/1472-6807-7-12)
Supplement: Additional file 1 — PFF01 Electrostratics. Full description of the electrostatic model used in PFF01. [file 1472-6807-7-12-S1.pdf]

PFF01 Electrostatics

The atoms of the protein are classified into potential types according to their chemical charateristics as described in[1]. The partial charges $q_i$and the screening constants D_{ij} were taken from an adapted version of an established electrostatic parameterization for proteins [2, 3]. The electrostatic energy is defined as

$$E_{lm} = f\ G_{lm} A_l A_m \sum_{i \in l, j \in m} \frac{q_i q_j}{r_{ij}}$$

where q_i is the partial charge on atom i and $r_{ij}$is the distance between atom i and j and f = 332.150625 kcal/mol. $A_l$is the relative surface area of group l and is defined by

$$A_l = \begin{cases} \frac{4}{S_{max}} \sum_{i \in l} S_i & (l \in \text{charged group}) \\ 1.0 & (\text{otherwise}) \end{cases}$$

where $S_i$is the exposed surface area of atom i. The values for $S_{max}$for the charged groups are shown in Table 2.
$G_{lm}$are the parameters based on groups l and m. The groups $l$and m are determined on the basis of Table 3.

and values of $G_{lm}$are given in the Table 4.

References

[1] T. Herges and W. Wenzel. An All-Atom Force Field for Tertiary Structure Prediction of Helical Proteins. *Biophys. J.*, 87(5):3100–3109, 2004.

[2] F. Avbelj. Use of a potential of mean force to analyze free energy contributions in protein folding. *Biochemistry*, 31:6290–6297, 1992.

[3] F. Avbelj and J. Moult. Role of electrostatic screening in determining protein main chain conformational preferences. *Biochemistry*, 34:755–764, 1995.

Table 1: Partial charges in PFF01. Only atoms with partial charges are listed. All other partial charges are zero.

Amino acid	partial charges	Net charge (e)
ASP	cb(-0.2), cg(0.34), od1-od2(-0.57)	-1
GLU	cb(0), cg(-0.2), cd(0.34), oe1-oe2(-0.57)	-1
HIS	cb(0.11), cg(0.17), nd1-ne2(-0.5), hd1-he2(0.37), cd2(0.33), ce1(0.65)	+1
LYS	cb-cg(0), cd(0.12), ce(0.3), hza-b-c(0.36), nz(-0.5)	+1
ASN	cb(0), cg(0.38),od1(-0.38), nd2(-0.56),hna-hnb(0.28)	0
PRO	n(-0.42), ca(0.21), cb(0), cg(0), cd(0.21)	0
GLN	cb-cg(0), cd(0.38), oe1 (-0.38), ne2(-0.56), hna-hnb(0.28)	0
ARG	cb-cg(0), cd(0.19), ne-nh1-nh2(-0.5), hne-hha-hhb-hhc-hhd(0.37), cz(0.46)	+1
SER	cb(0.03), og(-0.38), hog(0.35)	0
THR	cb(0.03), og1(-0.38), hog(0.35), cg2(0)	0
TRP	cb,cg,cd1,cd2,ce2,ce3,cz2,cz3 ,ch2(0), ne1(-0.28), hne(0.28)	0
TYR	cb,cg,cd1,cd2,ce1,ce2(0), cz(0.03), oh(-0.38), hoh(0.35)	0

Table 2: Charged groups and their maximum surface areas.

Charged Groups	Maximum SASA(S_{max}) $\mathring{A}^2$
ARG	168
ASP	105
GLU	117
HIS	143
LYS	135

Table 3: Group types based on amino acid types

Electrostatic Group type	Group types
CHR	EARG, NEH, CZ, ARG, ASP, GLU, HIS, NDH, NEH, LYS, PYP, FDS, BTN, AMAP, CFP
COS	COC, COD, COG, COS, ECD
COM	CO, ECO1, ECO2, ECO3, CO1, CO3, CO8, CO9, CO10, CO22, FDC
OH	NOH2, NOH3, NOH4, NOH6, OGH, OGS, OGT, OHH, O1H, O3H, OH6, OH10, OH12, OH13
NHS	C7N, NH2, NEW, NE2, ND2, FNA2, NC7, BEN
NHM	NH, ENH1, ENH2, FN10, ANH
NIL	N1, ACE, END, NTM, N-M, NA, XL, PRO

Table 4: Values of parameters G_{lm}

	NHM	COM	OH	NHS	COS	CHR	NIL
NHM	0.375731	0.375731	0.000000	0.143396	0.143396	0.043222	0.000000
COM		0.375731	0.161852	0.143396	0.143396	0.031012	0.000000
OH			0.000000	0.000000	0.161852	0.045452	0.000000
NHS				0.143396	0.143396	0.043222	0.000000
COS					0.143396	0.031012	0.000000
CHR						0.025000	0.000000
NIL							0.000000
